# Changes in late-latency auditory evoked potentials after tinnitus suppression using auditory stimulation

**DOI:** 10.1016/j.bjorl.2022.09.005

**Published:** 2022-10-12

**Authors:** Nastaran Ranjbar, Ali Shahbazi, Hossein Namvar Arefi, Navid Noori Zade, Mohammad Ali Nazari, Sadegh Jafarzad

**Affiliations:** a Department of Neuroscience, Faculty of Advanced Technologies in Medicine, Iran University of Medical Sciences, Tehran, Iran; b Cellular and Molecular Research Center, Iran University of Medical Sciences, Tehran, Iran; c Department of Audiology, School of Rehabilitation Sciences, Iran University of Medical Sciences, Tehran, Iran; d Otorhinolaryngology-Head and Neck Surgery Department, School of Medicine, Imam Reza Educational Hospital, Mashhad University of Medical Sciences, Mashhad, Iran; e Department of Audiology, School of Paramedical Sciences, Mashhad University of Medical Sciences, Mashhad, Iran

**Keywords:** Tinnitus, Auditory evoked potentials, P300, Residual inhibition, Tinnitus masker

## Abstract

**Objective:**

Tinnitus can result from auditory system reorganization due to neural activity dysfunctions. Auditory stimulation can cause temporary or persistent tinnitus alleviation by altering the neural generators. The present study investigated the changes in Late-Latency Auditory Evoked Potentials (LLAEPs) after tinnitus suppression using auditory stimulation with short-term Residual Inhibition (RI) and long-term Tinnitus Masker (TM).

**Methods:**

The study included 40 participates with equal numbers in the Tinnitus Group (TG), including the chronic tinnitus subjects, and the Control Group (CG), including matched volunteers. The participants had normal hearing in conventional audiometry. All the participants underwent LLAEP recording pre-intervention and after a one-minute auditory stimulation (RI), as well as a pre-intervention Dichotic Digit Test (DDT) as a behavioral assessment of the selective attention. Moreover, TG underwent a 3-month course of TM, a third LLAEP recording post-TM, a second DDT post-TM, and the Tinnitus Handicap Inventory (THI) before and after the TM.

**Results:**

In the baseline LLAEP recording, the TG had significantly later N1, P3a, and P3b latencies, as well as significantly lower P3a and P3b amplitudes. The second LLAEP recording showed a significant intragroup increase in P3a amplitude and a significant decrease in P3a latency in the TG, while no significant intragroup difference was observed in the CG. In the third LLAEP recording performed on TG, the P3a amplitude and latency had significant changes compared to the second recording, while the N1 latency was significantly decreased. Moreover, the DDT and THI scores had significant improvement after the TM in TG.

**Conclusions:**

The present study showed the neurophysiological changes after short-term (RI) and long-term (TM) auditory stimulations in tinnitus subjects. The LLAEP changes suggest that these interventions lead to tinnitus suppression through common mechanisms. The electrophysiological observation was also confirmed using behavioral assessments.

**Level of evidence:**

This study type is a “comparative study” with the level of evidence “3”.

## Highlights

• Late-latency auditory evoked potentials changes after tinnitus suppression.
• The N1, P3a, and P3b were significantly changed in tinnitus subjects.
• The P3a changes after short-term auditory stimulation in tinnitus subjects.
• The N1 latency decreases after long-term use of tinnitus masker.
• Our findings supports a common mechanism for residual inhibition and tinnitus masker.

## Introduction

Subjective tinnitus can be defined as an interpretation of sound without external auditory stimulus. Moreover, 10%–15% of the adult population has chronic tinnitus, of which 1%–3% are under tinnitus-induced psychological distress, impacting their quality of life.^1^, ^2^ One important clinical manifestation in tinnitus, is attention deficit. Attention can be defined as the ability of the simultaneous performance of different activities, and it has a crucial role in the generation and modulation of tinnitus.^3^, ^4^ It includes two distinct processes involving bottom-up and top-down processing. The interference between these two processes can lead to persistent tinnitus, resulting in neural dysfunction.^5^ This may lead to disturbed neural synchrony and subsequent neuroplasticity.^6^, ^7^ The association between attention and/or cognitive dysfunction with tinnitus has been investigated using electrophysiological, behavioral, and imaging studies.^3^, ^8^ Despite extensive research, the related pathophysiology is not illustrated, and the treatment primarily focuses on the subjective symptoms and clinical findings.^9^, ^10^

The changes in neuroplasticity and cognitive dysfunctions in tinnitus subjects can be investigated using the Late-Latency Auditory Evoked Potentials (LLAEPs).^8^ LLAEPs recorded using speech stimuli can be used for investigating the bottom-up and top-down processing and revealing the cerebral areas with altered cortical activity.^11^, ^12^ The most known late auditory evoked components are N1, P2 and P300. The P300 amplitude shows the speed of cognitive information processing,^12^ while its latency indicates the degree of attention.^13^, ^14^ Based on the test instructions, the P300 is recorded in two forms: P3a and P3b. P3a emerges from the passive hearing and correspond to bottom-up processing. On the other hand, P3b is recorded while the subject is attentively listening to target stimuli and is an indicator of top-bottom processing. Although most studies reported that LLAEPs tend to change in tinnitus subjects with normal hearing, there is a controversy in the altered components reported.^11^, ^15^, ^16^, ^17^, ^18^ Other diagnostic tool which evaluate attention dysfunction in subjects with tinnitus is the free recall form of the Dichotic Digit Test (DDT). DDT is easy to perform and can be used as a reliable tool in individuals with normal hearing and those with hearing loss for assessment of selective attention. However, the studies on DDT have yielded controversial results. In general, the presence of cognitive changes in subjects with tinnitus is controversial. These controversies can be explained by different study designs,^15^, ^19^ cognitive measures,^20^, ^21^ and participants' characteristics,^11^, ^14^ as well as the effect of covariates, such as age,^17^, ^19^ and the different stimuli used in electrophysiological studies.^22^, ^23^

Tinnitus suppression occurs in response to even a short-term auditory stimulation in some subjects. This phenomenon is known as the Residual Inhibition (RI).^24^ It is believed that this phenomenon can be explained by reductions in the spontaneous activity of different stages of auditory pathway neurons.^24^ In addition to the RI phenomenon, long-term auditory stimulation, known as the Tinnitus Masker (TM), can also suppress tinnitus.^25^ In theory, the TM can alter the tinnitus-related activities by increasing the system noise, leading to reduced tinnitus.^26^ Subjects with partial or complete RI may benefit from TM because it can lead to a potential reorganization or synchronization in the neuronal activity of the brain.^24^ TM is widely used in clinical settings and investigating its pathophysiology and mechanism can help to find the best therapeutic method for tinnitus. In order that, we hypothesize LLAEP changes recorded by the speech material after auditory stimulation and behavioral assessments may reflect the neurophysiological mechanism of tinnitus suppression. Therefore, we performed a baseline and two post-intervention LLAEP recordings and compared the results. The post-intervention assessments were performed after two interventions used for tinnitus suppression, including a one-minute auditory stimulation, which leads to the RI phenomenon, and a TM course lasting three months. Also, the DDT was used as a behavioral assessment of selective attention, and the Tinnitus Handicap Inventory (THI) was used for assessing the level of distress induced by tinnitus and its relationship with electrophysiological findings. To consider the effects of covariates, young volunteers with matched characteristics and normal hearing were included in the study.

## Methods

### Participants

The study included 40 adult participants divided into two groups of the Tinnitus Group (TG) and the Control Group (CG). The TG included 20 subjects with chronic tinnitus for at least 6-months^27^ with THI scores between 18–36 (mild handicap)^28^ and complete RI. The inclusion criteria in CG were 18–60 years of age with no history of acute or chronic tinnitus or other neuro-otologic disorders. Also, inclusion criteria for both groups were normal hearing,^29^ right-handedness, non-musician, mono-lingual,^30^, ^31^, ^32^ and non-depressed based on Beck Depression Inventory-II (BDI-II).^33^ Subjects with a history of using anti-depressant, anti-epileptic or ototoxic drugs were excluded from the study. Two study groups were matched based on age, and sex.

The Ethics Committee of the Iran University of Medical Science approved that the study protocol followed the ethical principles of the Declaration of Helsinki (Approval code: IR.IUMS.REC.1396.03.87.31978). All the participants signed the written testimonial before the participation and were fully informed of the study content.

### Procedure

**Figure 1:**
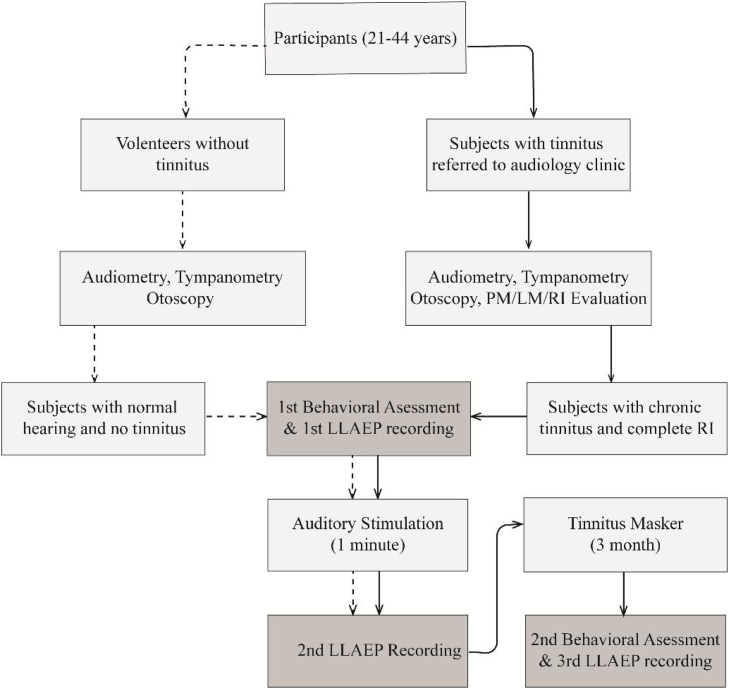
Flowchart of the study protocol. The dashed and solid lines show the study protocol step by step, including the enrollment, data collection, and data analysis for each group. CG, Control Group; TG, Tinnitus Group; LLAEP, Late-Latency Auditory Evoked Potentials; RI, Residual Inhibition; TM, Tinnitus Masker.

The study protocol is presented in Fig. 1. The psychoacoustic evaluation was performed to identify the Pitch Match (PM), Loudness Match (LM), RI, and the Minimum Masking Level (MML).^34^ The participants in the TG were also instructed to set the volume of the tinnitus masker at a level equal to their tinnitus loudness, which was the mixing point.^35^ For ensuring the appropriate use of the TM, participants completed a daily listening checklist that was proofed by one of the authors.

### Audiological evaluations

The audiological evaluation mentioned above included a detailed clinical history taking and examinations using otoscopy (Welch Allyn, model 25020A, USA), tympanometry (Inventis, model: Clarinet, Italy), and audiometry (Grason Stadler company, model: Pello, USA). Audiometry was performed at octave and mid-octave band frequencies of 125–8000 Hz in a standard acoustic room.

### LLAEP recording

**Figure 2:**
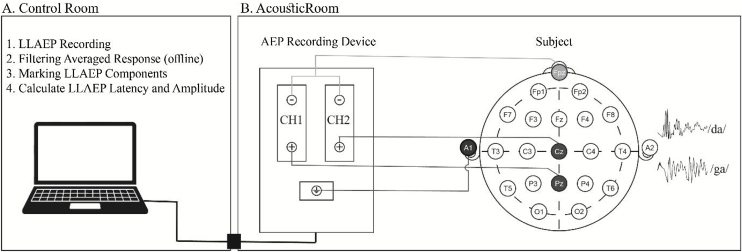
Schematic setup for LLAEP recording. (A) The examiner in the control room executes the recording and analyses the responses. (B) LLAEP, Late-Latency Auditory Evoked Potentials.

The participants were instructed to have enough rest and avoid smoking, caffeine, and extreme mental or physical activities for at least 24 h before the recording.^36^ Based on the 10–20 international system, Cz, Pz, Fpz, and A1 or M1 locations were prepared using a 70% alcohol solution and skin preparation gel (Weaver Company, Nuprep®, USA). Then, Ag-AgCl disposable electrodes (Leonhard Lang Company, Skintact®, Austria) were placed at the sites of interest shown in Fig. 2.

**Figure 3:**
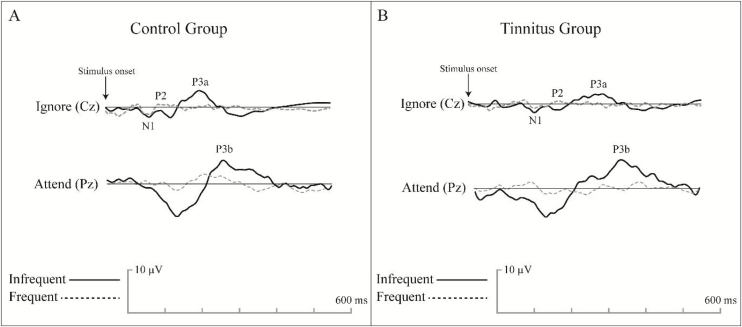
An example of the grand average LLAEP waveforms recorded from the control group (A) and tinnitus group (B) in the first LLAEP recording. The time window for the LLAEP recording was set at 600 ms, and the pre-stimulus duration was considered 100 ms. The amplitudes were calculated from the baseline to peak, while the latency was considered as the time at a defined peak in recorded time windows.

Electrode locations were first prepared, and then LLAEPs were recorded in a standard acoustic room with dim light. Two non-inverting electrodes were placed at Cz and Pz locations to record the responses in two separate channels. The inverting electrode was a linked electrode located at the left earlobe (A1) or Mastoid (M1). The ground electrode was placed at the Fpz. Impedance was kept below 5 KΩ for every single electrode, and the inter-electrode impedance was set at less than 2 KΩ. A 170-ms, frequent /da/, and infrequent /ga/ stimuli with a rate of 1.1 per second were presented unilaterally to the right ear via a supra-aural headphone (Telephonics, TDH39, USA). The participants were randomly exposed to 240 frequent (80%) and 60 non-frequent (20%) stimuli at the most comfortable level. For N1, P2, and P3a recording, they were asked to listen to the coming sounds (passive hearing). Then, they were instructed to push a button with their dominant hand as soon as they hear the infrequent /ga/ stimulus while ignoring the frequent /da/ stimulus with their eyes closed during the test to avoid eye-blinking artifacts. The recording was performed using a two-channel auditory evoked potential device (Neurosoft, Neuro-Audio, Russia) in a standard acoustic audiometry room with dim light. The participants attended a training session before the test. Fig. 3 presents a sample of recorded LLAEP responses in the CG and TG.

### Behavioral assessments

All the participants underwent behavioral assessment of the selective attention before the intervention using the free recall form of the DDT.^37^ DDT is an convenient and fast test and can be used for various age groups.^38^ Moreover, the TG underwent another assessment using the THI at the baseline and the end of the study duration.

## Results

### Audiological evaluations

Both groups were matched in gender (12 females and 8 males in each group) and had no significant difference in age (*t* = −1.092, *p* = 0.282). According to audiometry results, both groups had bilateral normal hearing (*p* =  0.241). 8 participants of the TG had unilateral tinnitus in their right ears, while 12 had bilateral tinnitus. Psychoacoustic evaluation results of the TG showed a PM range of 500–8000 Hz. Moreover, the mean RI duration in TG was 21.50 ± 4.58 min. Other socio-demographic characteristic of subjects is presented in Table 1.

**Table 1:** Socio-demographic profile of the study subjects.

Variable	Statistics	Tinnitus group (n = 20)	Control group (n = 20)
Sex (Males %)	n (Percent)	8 (40%)	8 (40%)
Age in year	Mean (SD)	32.65 (6.56)	30.50 (5.87)
Duration of tinnitus in months		13.65 (6.44)	‒
Pitch of tinnitus in KHz		4.05 (2.81)	‒
The loudness of tinnitus in dB SL		4.10 (1.11)	‒
Visual Analogue Scale		4.55 (1.60)	‒

### LLAEP recording

Statistical analysis showed that the Cz location had the optimal response (largest amplitude and shortest latency) for N1, P2, and P3a, while the Pz had the optimal response in the P3b. The mentioned finding was true for all the LLAEP recordings. Thus, the Cz was used for N1, P2, and P3a assessments, while the Pz was selected for the P3b assessment.

**Figure 4:**
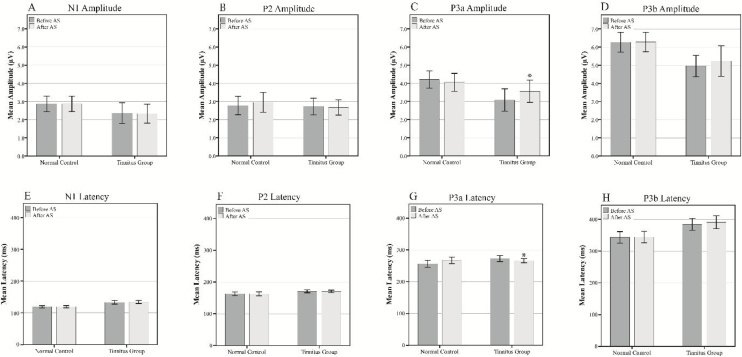
The mean amplitudes (A‒D) and latencies (E‒H) of the LLAEP components in the first and second recordings in the study groups. Significant changes are highlighted with asterisks. AS, Auditory Stimulation.

Inter-group comparisons of the LLAEP parameters of the first and second recordings are presented in Table 2. At the baseline recording, the mean P3a and P3b amplitudes were 3.07 µV and 4.95 µV in the TG, while they were 4.20 µV and 6.36 µV in the CG, respectively. Therefore, TG was significantly lower in these variables than the CG (P3a: *p* = 0.013, P3b: *p* = 0.002). The N1, P3a, and P3b latencies in TG was significantly later compared to the CG (N1: *p* = 0.000, P3a: *p* = 0.014, P3b: *p* = 0.002). In terms of intragroup differences between the first and second recordings, the only significant differences observed were the P3a amplitude and latency in the TG. The mean P3a amplitude in the TG increased from 3.07 µV to 3.55 µV (*p* = 0.001), while its mean latency 6.78 ms reduced (*p* = 0.017). The intragroup comparisons of the LLAEP parameters between the first and second recordings are presented in Fig. 4.

**Table 2:** Intergroup and intragroup comparisons of the mean amplitudes and latencies of the LLAEP parameters.

			Tinnitus group			Control group			Statistical result		
	Component		Mean	SD	n	Mean	SD	n	F	*p*	Cohen’s d^a^
Before	N1	Amplitude	2.36	1.14	18	2.87	0.86	18	1.116	0.214	0.422
		Latency	133.08	11.58	20	119.1	8.9	17	5.109	0	−1.55
	P2	Amplitude	2.72	0.89	17	2.77	0.98	17	0.306	0.871	0.056
		Latency	170.55	8.87	18	162.74	13.09	20	0.699	0.108	−0.535
	P3a	Amplitude	3.07	1.19	17	4.2	0.91	17	0.166	0.013	0.901
		Latency	273.08	19.67	18	256.67	23.56	20	0.06	0.014	−0.838
	P3b	Amplitude	4.95	1.15	17	6.26	1.08	18	0.188	0.002	1.169
		Latency	384.36	39.48	20	343.43	38.55	20	0.056	0.002	−1.049
After	N1	Amplitude	2.33	1.05	18	2.87	0.85	18	0.423	0.181	0.455
		Latency	134.44	40.12	20	119.37	6.96	17	0.904	0	−1.786
	P2	Amplitude	2.67	0.82	17	2.95	1.05	17	0.713	0.408	0.287
		Latency	169.27	8.94	18	162.68	13.66	20	2.426	0.091	−0.564
	P3a	Amplitude	3.55	1.18	17	4.05	0.96	17	0.945	0.293	0.367
		Latency	266.3	13.42	18	267.04	21.91	20	6.793	0.902	0.041
	P3b	Amplitude	5.22	1.62	17	6.27	1.08	18	7.855	0.021	0.816
		Latency	390.92	43.95	20	344.44	38.96	20	0.366	0.001	−1.119

a Cohen’s d was calculated as a measure of the effect size. Small effect = 0.2, medium effect = 0.5, Large Effect = 0.8.

**Figure 5:**
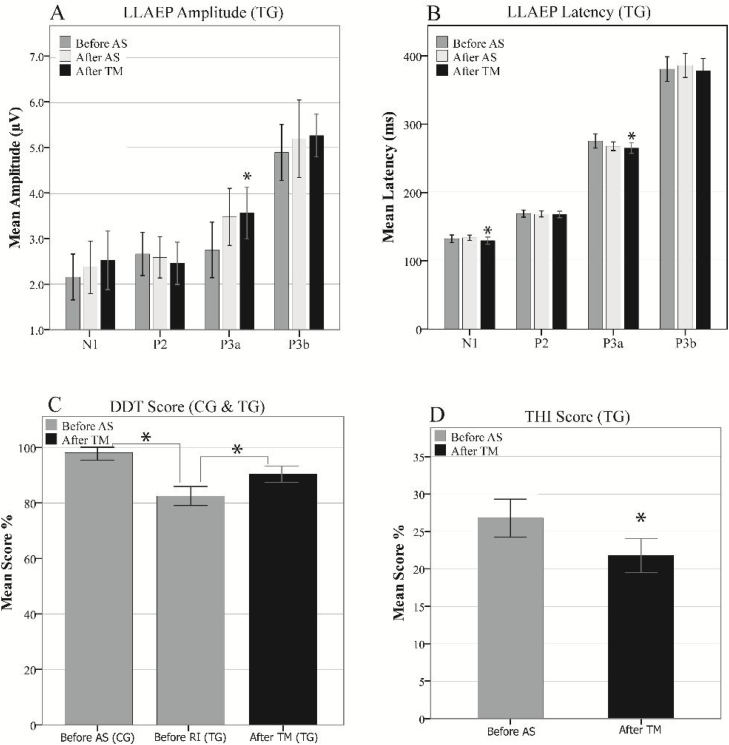
The mean amplitudes (A) and latencies (B) of the LLAEP components in the third recording in TG. C and D represent the mean scores for THI and DDT, respectively. AS, Auditory Stimulation; TM, Tinnitus Masker; THI, Tinnitus Handicap Inventory; DDT, Dichotic Digit Test.

At the third LLAEP recording, which was only performed in the TG following the TM course, the P3a amplitude and latency were significantly changed (*p* = 008 and *p* = 001, respectively), while there was also a significant decrease in the N1 latency (4.25 ms, *p* = 0.025) compared to the first recording. Moreover, there were insignificant increases and decreases in the P3b amplitude (*p* = 0.063) and latency (*p* = 0.059), respectively. However, P2 amplitude and latency did not differ significantly in the intra- and inter-group comparisons (Fig. 5A and B).

### Behavioral assessment

DDT scores of the baseline assessment were 98% and 82.52% in the CG and TG, respectively, with a significant difference between the groups (*t* = 5.26, *p* = 0.000). Moreover, the score increased significantly (7.85%) in the post-TM assessment in the TG compared to the baseline assessment (*t* = −12.53, *p* = 0.000). In terms of the THI scores, TG had a mean THI score of 26.67 ± 5.31 in the baseline assessment, which decreased to 21.78 ± 4.59 in the post-TM assessment, indicating a significant difference (*t* = 3.99, *p* = 0.001), as presented in Fig. 5C and D.

## Discussion

Suppression of tinnitus in response to auditory stimulations, such as complete RI or TM, has been observed in some subjects with tinnitus clinically; however, the underlying mechanism is not still well illustrated. The present study investigated the changes in the LLAEPs following tinnitus suppression induced by auditory stimulations. The reason we compared the short-term (complete RI) and long-term (TM) intervention for tinnitus suppression was the fact that the duration of tinnitus suppression will increase with prolonging the auditory stimulation.^39^, ^40^ Our results showed the N1 latency was significantly higher in the TG than the CG. The P3a and P3b amplitudes and latencies were significantly decreased and increased, respectively in the TG compared to the CG. Also, in first recording, P3a characteristics differed significantly between groups and in the third LLAEP recording, there were P3a alterations, as well as decreased N1 latency. As well, behavioral assessments confirmed our electrophysiological findings.

Any disruption in the top-down and bottom-up cerebral processing could lead to attentional disturbances.^41^, ^42^ In the baseline LLAEP recording, in TG, the N1 latency was significantly higher than the CG. The N1 response is emerged by attentive listening to a sound.^43^ This finding is compatible with the attentional disturbances observed in the behavioral assessments of the TG in our study. Moreover, the findings are consistent with other studies.^14^ However, there are controversies between the studies in the changes of the N1^3^, ^19^, ^44^ and P3b^11^, ^14^, ^18^ latencies in the subjects with tinnitus, which can be explained by different eligibility and selection criteria in the studies.

The P3a latency is an indicator of the time needed to start a cognitive task.^14^ Increasing the P3b latency is associated with disturbances in the classification speed or the time required for stimulus analysis.^12^ The P3a and P3b amplitudes were significantly decreased, while their latencies were increased in the TG compared to the CG. The increased amplitudes of P3a^14^ and P3b^13^ are due to attentional resource allocation. Moreover, the changes in the P300 amplitude are controversial in different studies.^15^, ^45^ The significant decrease in the P3a and P3b amplitudes in TG could be explained by the decreased number of neurons or neural activity and increased desynchrony.^45^ Although the P3b alterations in the second and third LLAEP recordings in TG were not significant, it shows a decreased time of cognitive processing.

There were significant intergroup differences in the P3a characteristics in the first recording. However, this difference became insignificant in the second recording, immediately after the short-term auditory stimulation resulting in the complete RI. This reflects that the required attentional sources for switching to the other salient increased immediately after the tinnitus suppression. In the third LLAEP recording performed in the TG after a 3-month course of TM, there were P3a alterations, as well as decreased N1 latency, suggesting a common mechanism for RI and TM and also improvements in the higher-order functional processing in the different regions of the cerebral cortex after the TM.

Electrophysiological studies can illustrate cerebral function, while the interaction of a set of cerebral networks can be evaluated by behavioral assessments. Our results on the behavioral assessments using DDT and THI after a 3-month course of TM confirmed our electrophysiological findings on the effects of long-term TM. Studies have controversial results on the DDT findings in tinnitus subjects. Some studies indicated that the dichotic auditory tasks were generally impaired in these individuals,^44^ while others reported no difference in selective attention.^20^ However, in our results, the baseline DDT score was significantly lower in the TG compared to the CG, while it significantly improved after the TM course. This was also true for the THI score, suggesting the clinical advantage of this intervention in TG.

The Cz electrode location is suggested for recording the N1-P2 and P3a components, while the Pz is reported to have the highest amplitude for P3b.^46^ In the present study, we used these locations for LLAEP recording, and it was compatible with the previous studies. We used a two-channel LLAEP recording system due to the following reasons: (1) Acquiring the optimal amplitudes and latencies for the desired LLAEP components, (2) Minimizing the measurement noises, which is a limiting factor in the high-resolution EEG systems,^47^ (3) Decreasing the setup time and computation requirement,^48^ (4) Reducing the signal bandwidth,^48^ and (5) The availability of the equipment and its clinical advantage. The present study was conducted on a homogenous group of subjects to avoid the possible effects of confounding factors, such as hearing loss, age, right- or left-handedness, depression, and tinnitus characteristics.

Several studies have reported the speech processing disturbances at competing listening conditions in tinnitus subjects,^49^, ^50^ even in individuals with normal hearing.^51^, ^52^ Speech stimulus material is used for LLAEP recording because its processing requires a higher-order function of the brain. Therefore, it can better predict the neuroplastic impairments and changes in subjects with chronic tinnitus than simple pure tone signals.^53^, ^54^, ^55^ Our findings can help clinicians to monitor the theraphetic effects of the current treatment methods, and also suggests the potential beneficial effects of new cognitive therapy methods such as attentional training.

## Conclusion

Study results showed LLAEP changes following the tinnitus suppression induced by short-term (complete RI) and long-term (TM) auditory stimulations in subjects with chronic tinnitus, suggesting a common mechanism of tinnitus suppression for these two types of auditory stimulations. Moreover, there were changes in the behavioral assessments after these interventions that confirmed the electrophysiological findings.

## Funding

This research was supported by the Iran University of Medical Sciences (Thesis Number:1330).

## Conflicts of interest

The authors declare no conflicts of interest.
